# Supplementary material for: Are carbon emissions trading and green financial instruments synergistic? -Comprehensive quantitative research based on content analysis
Source: PLoS One. 2024 Mar 7;19(3):e0298601. doi: 10.1371/journal.pone.0298601 (PMC10919601; doi:10.1371/journal.pone.0298601)
Supplement: S1 Data — (ZIP) [file pone.0298601.s001.zip › Data/地级市Z_F工作报告.zip!/╣π╢1⁄2/╗▌╓▌╩╨/└┤╫╘╣1⁄2╓┌║┼í╛┬φ┐╦╩2╛▌═°í┐_2018.docx]

各位代表：

　　现在，我代表市人民政府向大会报告工作，请予审议，并请政协各位委员和其他列席人员提出意见。

　　2017年工作回顾

　　刚刚过去的一年，我们深入贯彻落实党的十八大、十八届历次全会和省第十二次党代会、市第十一次党代会精神，认真学习贯彻党的十九大精神，以习近平新时代中国特色社会主义思想为指导，在省委、省政府和市委的坚强领导下，在市人大、市政协的监督支持下，自觉践行新发展理念，坚持稳中求进、好中求快、改革创新、率先跨越，统筹做好改革发展稳定各项工作，经济社会保持健康较快发展，较好完成了市十二届人大一次会议确定的目标任务，建设绿色化现代山水城市实现良好开局。

　　（一）突出供给侧结构性改革，着力抓项目、强实体，经济实力持续增强。全年实现地区生产总值3830亿元，同比增长8%左右（预计，下同）；来源于惠州的财政总收入突破千亿大关，达1000.9亿元，增长21.8%；地方一般公共预算收入389.1亿元，增长10%。“去降补”任务有效落实。234家国有关停企业和4家非国有“僵尸企业”出清，95家国有特困企业脱困。完成压减50万吨精钢和淘汰61万吨落后钢铁产能任务。认真贯彻落实国家、省调控政策，房地产市场保持健康平稳发展。金融机构本外币存贷款余额分别为5485.6亿元、4012.9亿元，存贷比73.15%，不良贷款率0.93%，低于全省0.75个百分点。降低企业成本约162亿元。补短板工程完成投资173亿元。项目建设提速增效。全面推行首席服务官制度，230宗重点项目完成投资643.5亿元，完成年度计划的106.8%，117宗亿元以上项目建成投产。完成固定资产投资2240亿元，增长10%。新引进外资项目661宗，实际利用外资11.5亿美元。国内外市场不断拓展。成功举办惠州产品（福州）展销会、惠州产品（墨西哥）展览会、2017中国手机创新周暨第六届云博会。社会消费品零售总额1360亿元，增长11%。进出口总额3416亿元，增长12.2%；其中出口2233亿元，增长13.2%。服务企业成效明显。出台实体经济“惠十条”。设立全省首家省市共建的中小微企业发展基金，市中小微企业贷款风险补偿基金支持企业获得贷款30.9亿元。“小升规”企业821家，规模以上工业企业达到2171家。新增市场主体9.6万户，增长37.3%。新增A股上市企业4家、新三板挂牌企业11家。

　　（二）突出产业转型升级，着力优结构、提质量，产业竞争力持续提升。深入实施“产业培育计划”，“2+2+N”现代产业体系进一步完善，三次产业结构比例4.5∶55∶40.5。支柱产业加快向中高端发展。中海油炼化二期1000万吨炼油项目成功投产、120万吨乙烯工程竣工试车，TCL模组整机一体化项目动工建设，旭硝子第8.5代显示玻璃项目实现量产。先进制造业和高技术制造业增加值分别占规模以上工业增加值的62.2%和40.5%，居全省前列。完成工业技改投资430亿元，居全省第三。新增国家级智能制造试点示范项目4个。入选全省4K电视网络应用与产业发展试点市。现代服务业不断壮大。楼宇经济加快发展，软件和信息技术服务业收入215亿元，增长20.1%；电子商务交易额1700亿元，增长20%。全域旅游示范区创建扎实推进，全年接待游客5393.6万人次、旅游收入439.3亿元，分别增长14.4%、20.6%。西湖创5A级旅游景区有序推进。现代农业加快发展。累计建成现代农业示范基地120个，农业龙头企业、农民专业合作社分别达280家、2236家。新增国家地理标志农产品2个。农机化整体水平居全省前列。成功举办第五届农博会。

　　（三）突出创新驱动发展，着力筑平台、聚要素，发展动能持续释放。牢牢扭住创新驱动发展“八大抓手”发力使劲，启动国家创新型城市“十大工程”建设，创新生态不断优化。“海绵行动”深入实施。对深招商选资196宗，总投资2210.9亿元，其中引进高技术企业59家。深莞惠经济圈（3+2）第十次联席会议32宗涉惠合作事项有效落实，全面对标深圳成效明显。开放式创新平台取得突破。中韩（惠州）产业园获国务院批复设立。

　　珠三角（惠州）国家自主创新示范区空间发展规划编制完成。环大亚湾新区区域协同发展加快推进，111宗建设项目完成投资241亿元，三大起步区及 “1+N”创新产业集聚区加快建设，世界级石化产业基地初步形成。“广东硅谷”建设提速。潼湖生态智慧区思科科学城、碧桂园创新小镇等引领性项目动工建设，14家高端研发机构及平台落户，创新园初具规模。潼湖科技小镇入选省首批特色小镇创建示范点。创新主体加快壮大。净增高新技术企业336家，总量达802家。新增科技企业孵化器4家，总量达26家，孵化面积突破100万平方米。新增众创空间8家，总量达28家，金百泽云创工场获评国家级众创空间。净增新型研发机构6家，共认定15家。主营业务收入5亿元以上工业企业研发机构实现全覆盖。建成北斗开放实验室惠州分实验室。中科院“两大科学装置”顺利推进。设立诺贝尔奖获得者工作站1个，新增院士工作站2个，新组建省级工程中心59家。夏佳文院士重离子医用加速器团队、王复明院士工程安全与防护团队、TCL集团新型量子点显示技术团队入选“珠江人才计划”，共获得省项目专项扶持资金1.7亿元；亿纬锂能高性能锂离子电池产业化项目获国家发改委资助资金1.75亿元。创新成果量质齐升。PCT专利申请量350件，万人发明专利拥有量10.37件，获十九届国家专利优秀奖6项。38家企业通过知识产权“贯标”认证，新增国家知识产权优势企业2家。成功举办首届中国高校科技成果交易会、第十二届中国研究生电子设计竞赛全国总决赛暨全国智能硬件创新创业大赛、全国智能制造应用技术技能大赛、2017中国创新创业大赛军民融合专业赛等活动。

　　（四）突出区域统筹协调，着力夯基础、拓功能，城乡一体化持续推进。“一市六组团”发展格局加快形成，城乡面貌不断更新。城市建设扩容提质。启动新一轮土地利用总体规划、城市总体规划修编，入列国家“城市双修”试点，建成 “海绵城市”10平方公里。17条城市主次干道加速推进，连接四条城市主干道的金石二路建成通车。惠州大道东段延长线、芦泰大桥动工建设。惠东大岭、博罗罗阳和龙溪、龙门平陵四个镇“撤镇设街道”。大力推进节约集约用地，消化批而未供土地2.27万亩。“两港三网”加快推进。惠州机场新增6条航线，年旅客吞吐量95.7万人次，增长74%；机场路二期建成通车。惠州港荃湾煤炭集散基地一期投入运营，亚洲规模最大的华瀛燃料油调和配送中心及配套码头完工；新开通香港、上海航线，港口吞吐量7211万吨。5条在建高速公路加快推进。赣深高铁、广汕高铁惠州段动工建设。新增光纤接入用户52.3万户，光纤入户率108%、固定宽带普及率105%、光网覆盖率220%，均居全省前列。城市精细化管理效能提升。城市管理执法体制改革深入推进，在全省率先设置城市治理分院。“两违”整治力度不断加大，地面建筑物全面普查数据库初步建成，查处违法用地1091宗6164亩、违法建筑5159宗185万平方米。“惠民城管通”“惠民交通APP”服务平台进一步优化，“和美网格”应用获评“省电子政务优秀案例”。部分道路实施潮汐车道制，市区公交TC改革顺利实施。新农村建设强力推进。全面推行“3+3+X”模式，新农村示范片建设走在全省前列，150个省市贫困村示范村创建工作全面启动。农村土地确权登记颁证任务基本完成。村居公共服务站、农村普惠金融“村村通”实现全覆盖。“美丽乡村·三大行动”深入推进，农村基础设施不断完善，建成森林村庄162个，在全省率先完成“村村通”自来水工程，新建农村污水处理设施368个，完成自然村村道硬底化1252公里，基本实现行政村污水处理设施、通班车、通4G网络和自然村村道硬底化全覆盖。惠阳区获评“四好农村路”全国示范县（区）。

　　（五）突出绿色化引领，着力建机制、抓整治，生态优势持续彰显。践行“绿水青山就是金山银山”理念，获评首批国家生态文明建设示范市，生态城市健康指数全国第六，环境空气质量居珠三角第一、全国74个重点监测城市前列。创新生态环保管理机制。率先实行自然资源资产清单管理，构建GDP、GEP“双核算”体系，采用量化方法为生态环境打上“价格标签”，生态文明建设责任进一步强化。生态创建成效显著。市级生态村实现全覆盖，所有县区均达到国家生态文明建设示范县区创建标准。全年造林17.9万亩，建设生态景观林带2.2万亩、森林碳汇工程3.1万亩，改造和新建风景林625亩，完成水源林505亩，入选全省首批森林小镇2个，新增森林公园13个，建成古树名木信息管理系统。成功承办中国生态文明论坛 （惠州）年会，获评2017美丽山水城市、中国十大养生城市。大亚湾红树林城市湿地公园获评国家城市湿地公园，潼湖湿地公园获批国家湿地公园试点。污染防治扎实有力。全面落实河长制，加强淡水河、潼湖流域水环境整治，东江干流惠州段、西枝江水质保持稳定达标。市中心区河涌整治持续推进，望江沥、新开河综合整治基本完成；城市建成区27条黑臭水体整治工程全部开工，17条已整治达标；79条镇级河涌中64条完成年度整治任务。建成梅湖污水处理厂三期工程，完成淡水污水处理厂主体工程，新增城镇污水管网175公里。考洲洋生态修复工程加快推进，近岸海域一、二类水质达标率95.6%；建立土壤污染治理修复项目库，完成146个土壤监测点位布设。常态化开展大气污染防治，加大扬尘及臭氧污染防控，完成国华、平海电厂3台机组超低排放改造。环境监管力度加大。划定海洋生态红线，强化入海排污总量控制。严格环保准入，否决不符合环保要求项目135个，否决率10.1%。严格环保执法监管，立案处罚环境违法行为1345宗，高标准推进中央环保督察组、国家海洋督察组反馈意见整改落实。节能减排深入推进。完成污染减排重点项目50个，清退重污染项目14个，淘汰黄标车1024台，新增绿色建筑面积670万平方米。大亚湾石化区获评国家第一批绿色制造体系建设示范园区和国家循环化改造示范试点园区。

　　（六）突出两大核心任务，着力补短板、强弱项，全面小康指数持续提高。紧紧围绕全面建成小康社会、打赢精准脱贫攻坚战，集中力量编牢织密保障网，确保小康路上一个都不掉队。精准扶贫扎实推进。深入开展“三个一百”活动，全市500多个帮扶单位和7000多名党员干部与贫困村、贫困户挂钩对接，实现结对帮扶全覆盖。累计投入5.06亿元，实施村扶贫项目1116个，圆满完成省下达的年度脱贫任务，建档立卡贫困户38772人实现预脱贫。全面小康建设步伐加快。居民人均可支配收入3.1万元，增长10%。全面建成小康社会综合指数达97%，同比提升3个百分点。惠东高潭革命老区“建成三个基地，办好十件实事”扎实推进，高潭区苏维埃政府成立90周年纪念活动圆满成功。保障水平稳步提高。新增城镇就业7.5万人，创业带动就业2万人，在全省率先完成本市户籍人员社会保险参保登记工作。城乡低保标准和五保供养、孤儿生活保障标准分别提高到每人每月740元、1184元、1500元。贫困对象政策内住院医疗费用救助比例提高到100%。流浪乞讨人员托养服务管理和救治、安置工作更加规范。配租公租房5177套，发放租赁补贴1111户。棚户区改造安置房新开工1700户，建成452套。农村危房改造开工9505户，竣工8347户。

　　（七）突出全民共建共享，着力抓创建、办实事，民生社会事业持续进步。全市公共财政民生支出397.7亿元，增长10.8%，占一般公共预算支出比重的71.8%。省民生实事涉及我市的59项工作及市十件民生实事70项工作完成年度目标任务。文明创建实现新突破。高水平夺取全国文明城市“四连冠”，博罗、龙门新入选全国文明县城，惠东县成为全国县级文明城市提名候选城市。新增省级以上文明单位、文明村镇18个。成立志愿服务学院和志愿服务救护培训中心，注册志愿者53万多人。“中国好人”增至27人、“广东好人”增至37人。教育惠民水平不断提升。新办幼儿园58所，新增普惠性学前教育学位1万多个。新建和改扩建中小学27所，新增公办义务教育学位2.65万个。基础教育质量不断提升，高考本科上线率首次超过50%。惠州工程职业学院成功申办、顺利招生，省市共建惠州学院工作有序推进。清华大学等22所国内知名大学签约参与大学创新园建设，哈尔滨工业大学国际教育科研基地成功落户。文体事业蓬勃发展。入选国家文化消费试点市，基本公共文化服务标准化等4项省级试点任务全面通过验收，5个公共图书馆通过国家一级馆复评，发放文化惠民卡12万份，新建村（社区）综合文化服务中心约1000个。加强非物质文化遗产保护管理，东坡祠主体工程基本完工，宾兴馆抢修有序推进。《邓演达》《一缕阳光》等一批惠州特色文艺精品深受好评，《海上升明月》《记住乡愁》荣获省“五个一”工程奖。成功承办中华龙舟大赛、省第四届体育大会、首届全国青少年国防体育大会、粤港澳大湾区流行文化系列活动暨第三届粤港澳台微影视作品文化交流周。健康事业加快发展。扎实推进医疗卫生体制改革，卫生与健康“十大工程”顺利推进，市中心人民医院通过“三甲”复审，第一人民医院、第三人民医院成功创建“三甲”。成为全省首个公立医院薪酬制度改革试点市。建成医联体20个，累计建成75间基层中医馆，在全省率先建立村级中医馆服务模式。组建家庭医生式服务团队591个，家庭医生签约服务重点人群覆盖率64.6%，计生特殊家庭100%纳入签约服务。获评省妇幼健康优质服务示范市。成功承办第三届国际养生大会。社会治理成效明显。“平安细胞”创建率100%，扎实开展“飓风”“安网”等专项行动，深入推进全民禁毒工程，刑事发案数下降19%，“两抢”案件下降62%，“两抢”零发案天数创五年新高。连续三次获评全国社会治安综合治理优秀市，夺取全国综治最高荣誉“长安杯”。社会信用体系建设考核连续5年全省第一，获评全国首批社会信用体系建设示范城市。“七五”普法扎实开展，公共法律服务水平进一步提高。启动省级食品安全城市创建工作，468家企业纳入省食品安全追溯系统监管平台，43家粮油企业进入市粮油二维码溯源系统。严格落实安全生产责任制，全市安全生产形势稳定。

　　与此同时，我们进一步加强国防教育、人民防空、民兵预备役、双拥共建、复退军人服务等工作，支持驻惠部队深化改革，军民深度融合发展取得新进步。加强民族宗教工作，广东道教学院顺利开办。统计、物价、供销、口岸、打私、方志、档案、老龄、科普、气象、三防、消防、应急、移民、防震减灾、对口援建等工作取得新成效，港澳台、青少年、残疾人、红十字、老区建设等工作再上新台阶，各项社会事业全面进步。

　　（八）突出政府自身建设，着力推改革、转作风，服务效能持续提升。大力推进政府系统作风建设，促进部门转变职能、聚焦主业，政府公共服务总体满意度全省第一，政务环境满意度全省第二。严守政治纪律政治规矩。坚持学深做实、走在前列，把深入学习贯彻党的十九大精神和习近平新时代中国特色社会主义思想作为首要政治任务抓实抓好。严格落实中央八项规定精神，扎实推进“两学一做”学习教育常态化制度化。坚持重大事项向市委请示报告，严格落实 “三重一大”决策制度。全面推进政府系统党风廉政建设，行政监察、审计监督、督促检查力度不断加大。“放管服”改革深入推进。编制新一轮权责清单调整目录，取消60项、下放（委托）33项。投资项目审批制度系统集成改革成为省试点，“首席服务官”制度作全国经验交流。“一门式一网式”政务服务改革扎实推进，所有村居开通网上服务大厅，在全省率先完成村（社区）公共服务平台建设任务。公共资源交易改革深入推进，服务收费全面取消。政务服务数据共享交换体系初步建成，市级平台归集数据近9亿条。阳光政务建设取得实效，公开政务信息14.2万条。法治政府建设稳步推进。决策咨询和定期学法制度不断健全，市、县两级政府法律顾问制度全覆盖。坚持向市人大及其常委会报告工作，向市政协通报情况，主动自觉接受监督。提请市人大常委会审议地方性法规草案1件，制定政府规章2件、规范性文件19件。办理人大议案9件、代表建议170件、政协委员提案203件，办复率100%。

　　各位代表，过去一年，前行充满艰辛，成绩来之不易。这是省委、省政府和市委总揽全局、正确领导的结果，是市人大、市政协有效监督、大力支持的结果，是全市人民齐心协力、团结拼搏的结果。在此，我代表市人民政府，向辛勤工作在各行各业的广大干部群众，向大力支持政府工作的人大代表、政协委员、各民主党派、各人民团体和各界人士，向关心帮助地方发展的驻惠人民解放军、武警部队官兵、驻惠单位，向积极参与惠州建设的港澳台同胞、海外侨胞、国际友人，表示衷心的感谢和崇高的敬意！

　　成绩令人鼓舞，问题不容忽视。前进的道路上，我们还面临诸多困难挑战，政府工作还存在不少差距不足：有效应对经济下行压力，推动新常态下发展的办法还不够多，稳增长还需付出艰苦努力；产业结构有待优化，战略性新兴产业培育力度不够，现代服务业有待提质壮大；自主创新能力还不够强，高新技术企业、新型研发机构、科技企业孵化器与发达地区相比差距较大，人才总量特别是高层次人才还不能满足高质量发展的需要；国际化法治化市场化营商环境有待优化，与发展高水平开放型经济仍不适应；区域城乡发展不平衡不充分问题较为突出，统筹协调发展的任务依然艰巨；公共服务与群众新期待还有不少差距，保障和改善民生仍需做大量工作；违规用地、违法建设问题时有发生，城市建设管理、土地管理水平有待提升；影响社会稳定的风险隐患仍然不少，创新社会治理有待加强；政府职能转变、作风建设有待深化，执行能力、服务意识、办事效率有待进一步提高。对于这些困难和问题，我们一定勇于担当负责，努力加以解决。

　　2018年工作安排

　　2018年是贯彻落实党的十九大精神的开局之年，是改革开放40周年、惠州撤地设市30周年，是实施“十三五”规划、决胜全面建成小康社会的关键一年。今年政府工作的总体要求是：以习近平新时代中国特色社会主义思想为指导，全面贯彻党的十九大和中央经济工作会议、中央农村工作会议精神，贯彻落实省委十二届二次、三次全会精神，按照市委十一届历次全会、市第十一次党代会第三次会议的部署要求，坚持稳中求进、好中求快、改革创新、率先跨越，坚持新发展理念，紧扣社会主要矛盾变化，贯彻高质量发展的要求，统筹推进“五位一体”总体布局和协调推进“四个全面”战略布局，坚持以供给侧结构性改革为主线，深入实施创新驱动发展战略，加快构建开放型经济新体制，坚决打赢防范化解重大风险、精准脱贫、污染防治三大攻坚战，全力做好稳增长、促改革、调结构、惠民生、防风险、优环境各项工作，促进经济社会持续健康发展，奋力开创建设绿色化现代山水城市新局面，为广东建设成为向世界展示习近平新时代中国特色社会主义思想的重要“窗口”和“示范区”作出惠州新贡献。

　　——— 做好今年工作，必须增强率先跨越的信心。当前，我国正处于一个大有可为的历史机遇期。习近平新时代中国特色社会主义思想，让我们有了把握机遇的根本指南和深厚底气。我们要自觉把惠州放在全省、全国的发展大格局中来谋划发展，提高站位、找准定位，从全局出发抓工作、以全球视野配资源，坚定建设绿色化现代山水城市目标不动摇，抢抓“一带一路”、粤港澳大湾区、中韩（惠州）产业园建设等机遇，进一步做优产业结构，做强发展平台，做活生态文章，做实民生保障，努力开创建设绿色化现代山水城市新局面。

　　——— 做好今年工作，必须坚持走高质量发展之路。进入新时代，我国经济已由高速增长转向高质量发展阶段。我们要全面准确贯彻高质量发展的根本要求，统筹考虑稳与进、供与需、质与量的统一，该稳的要稳住，该进的要进取，把进的方向和目标落到提高发展质量和效益上，在改革创新、结构调整、动力转换等关键领域积极进取，推动惠州经济尽快进入高质量发展的良性轨道，实现经济发展质量变革、效率变革、动力变革，加快构建现代化经济体系。

　　——— 做好今年工作，必须弘扬真抓实干好作风。市第十一次党代会第三次会议作出了“五个牢牢把握”和“四个方面重点”的决策部署，为做好全年工作指明了方向、明确了路径。我们要积极传承“红船精神”和高潭革命精神，始终保持务实高效作风，坚持“马上就办、真抓实干”，践行“一线工作法”，强化督促检查，落实层级责任，以踏石留印的力度、抓铁有痕的韧劲，定一件干一件、干一件成一件，件件抓到底、件件出成效，在新时代干出新气象、推动新发展、实现新作为！

　　今年我市经济社会发展的主要预期目标是：生产总值增长8.5%左右；人均GDP增长7.5%左右；一般公共预算收入增长10%；固定资产投资增长10%；社会消费品零售总额增长11%；出口额增长5%；全社会研发经费支出占GDP比重2.65%（按可比口径）；居民消费价格累计增长控制在3%以内；全体居民人均可支配收入增长10%；城镇登记失业率控制在2.5%以内；常住人口自然增长率、节能减排等各项指标完成省下达任务。

　　实现以上预期目标，重点抓好十方面工作：

　　（一）深化供给侧结构性改革，推动经济高质量发展迈出新步伐

　　着力扩大有效投资。全年安排重点项目247宗，年度计划投资634.3亿元，切实把有效投资转变为有效供给。用好发展实体经济推动项目建设现场会成果，着力提高履约率、开工率、竣工率和投产率，推进省市重点项目和新立项亿元以上项目落地建设。加大工业投资力度，力争工业投资800亿元以上。抓好SMPO/POD项目建设，科学谋划炼化三期项目，拓展石化中下游及关联产业。加快TCL模组整机一体化、伯恩光学新基地、旭硝子二期等电子信息产业项目建设，推动欣旺达二期、科达利等新能源项目建设。加强与世界500强、大型企业务实合作，引进一批产业带动强、科技含量高、税收贡献大的优质项目。

　　大力推进 “惠州智造”。全面落实《中国制造2025》，推动产业向中高端发展，提高供给体系质量和效率。加快发展智能终端产业、先进装备制造业，大力推进东风本田一体化、亿纬锂能新能源产业园等项目，加快培育新能源汽车整车和关键零部件骨干制造企业，推动企业由生产智能产品向生产智能装备拓展。积极发展核电、风电、太阳能、LNG等清洁能源产业。深化制造业与互联网融合发展，鼓励企业使用智能化制造设备，推广机器人应用。抓好中国信息通信研究院华南创新基地、赛迪（华南）智能制造创新中心建设。实施新一轮工业技改三年行动计划，引导400家以上企业开展技术改造。大力弘扬工匠精神，实施名牌带动战略，扩大名牌产品集群，争创全国质量强市示范城市。

　　加快打造“数字惠州”。紧跟国家实施大数据战略，不断完善大数据基础设施，推动数据资源整合和开放共享，以数字政府建设带动数字惠州建设。推行“互联网+园区”发展模式，抓好潼湖生态智慧区省级重点大数据产业园建设。支持工业互联网平台建设，助推企业“登云触网”。积极拓展信息消费新产品、新模式，扩大信息消费覆盖面。推动5G技术创新应用与产业发展，支持企业参与技术标准制定。深入开展新数字家庭行动，推动4K应用示范社区（村）建设，加快发展4K电视网络应用与产业。办好2018中国手机创新周暨第七届云博会。

　　提高现代服务业规模和质量。着力培育总部型、创客型楼宇经济，推动电子商务、会展经济发展壮大。加快发展现代金融业，深化农商行改革。加快现代物流园区建设，强化供应链服务，发展现代物流业。积极发展文化创意、研发设计、融资租赁、服务外包等生产性服务业。扎实推进全域旅游示范区创建，抓好西湖创5A级旅游景区、巽寮创国家级旅游度假区，打造罗浮山—南昆山百里生态景观长廊和惠东－大亚湾百里滨海旅游景观带。出台康养产业发展规划，推动康养产业集聚化、规模化、高端化发展。开展扩大消费增品种、提品质、创品牌专项行动，打造一批绿色商场。

　　激发各类市场主体活力。依法保护各类所有制企业合法权益，激发和保护企业家精神。培育大型骨干和后备骨干企业，推进“小升规”“个转企”，形成各类企业梯次发展格局。贯彻落实好省、市“实体经济十条”，降低生产要素成本和物流成本。分类推进国有企业改革，促进国有资产保值增值。深化企业服务月活动，优化升级“惠企通APP”和微信公众号，打造“首席服务官”升级版。加大工业用地盘整力度，帮助企业解决用地困难。完善推动金融服务实体经济政策体系，大力实施企业上市倍增计划。充分发挥中小微企业发展基金、贷款风险补偿基金和政策性融资担保机构作用，降低企业融资成本。做好全国第四次经济普查工作。

　　（二）深入实施创新驱动发展战略，推动国家创新型城市建设实现新突破

　　大力推进创新平台建设。推动珠三角 （惠州）国家自主创新示范区建设提速，出台先行先试政策。推进仲恺高新区“357创新产业带”建设，发展云计算、物联网、先进装备制造等战略性新兴产业。建设国家北斗终端产品质检中心。抓好潼湖生态智慧区基础设施建设，推进思科潼湖科学城、碧桂园创新小镇、哈工大国际教育科研基地、大学创新园建设，打造“广东硅谷”。规划建设罗浮新城。

　　培育壮大创新主体。开展“高企树标提质行动”，加快高新技术企业培育，确保高企总量1000家以上。瞄准世界科技前沿，着力推进中科院“两大科学装置”、比利时广东IMEC研究中心等重大项目建设。支持企业与高校、科研院所共建研发机构，力争规模以上工业企业设立研发机构比例达40%以上。实施孵化育成体系提质增效行动，孵化面积120万平方米以上。强化知识产权创造、保护、运用，筹建国家级知识产权保护中心，万人发明专利拥有量11件以上。

　　优化创新创业生态。办好第二届科交会，发挥好中国（华南）高校科技成果转化中心作用，促进科技成果转化和产业化。推进军民深度融合发展，引进更多国防科技成果到惠落地。落实股权和分红激励等政策，推进产学研合作。搭建大众创业、万众创新平台，大力开展“双创”活动，激发全社会创新活力。鼓励知识产权质押融资，大力发展和引进风险投资、股权投资，促进金融与科技有机融合。大力实施“人才双高计划”“人才双十行动”，引进领军人才20名以上、高水平创新团队10个以上。建设一批海外人才工作站，建成高层次人才“一站式”服务窗口。着力解决人才落户、住房、子女入学、出入境等问题，让人才引得进、用得好、留得住。

　　加快实施“海绵行动”。全面对接深圳“东进”战略，推动“深圳研发、惠州产业化”，主动吸纳深圳优质创新资源，加快引进一批掌握自主知识产权、创新带动能力强的研发型企业。积极对接广深科技创新走廊，打造百里高铁创新产业带；推动共建珠三角高新技术企业数据库及培育后备库，共建科技企业孵化器、众创空间。深入实施珠三角规划纲要，认真落实深莞惠“3+2”对接事项，加强与东莞、汕尾、河源互补合作，推动实现协同发展。

　　（三）加强对外交流合作，推动构建开放型经济新体制取得新进展

　　积极参与粤港澳大湾区建设。落实粤港澳大湾区城市群发展规划，推动基础设施互联互通，加快融入大湾区1小时都市圈。探索与香港、深圳等地共建产业园区，实现优势互补、资源共享。强化科技创新合作，拓展仲恺港澳青年创业基地功能，建设粤港澳大湾区科技成果转化高地。落实《粤港澳大湾区卫生与健康合作框架协议》，构建大湾区卫生与健康共同体。加快打造环大亚湾开放型现代化新区，扎实推进对接先进地区的发展通道、产业承接平台以及45宗现代产业项目建设，提升三大起步区建设水平。

　　全力建设中韩（惠州）产业园。积极争取国家、省出台支持产业园发展的政策措施，完善《中韩（惠州）产业园总体规划》，加快制定建设实施方案，建立健全管理机构。编制对韩招商图谱，加大对韩招商推介，促进韩国高端要素在惠州聚集。鼓励支持有条件的企业到韩国新万金投资兴业，扩大双向投资规模。争取承办第七届韩国—广东发展交流会。充分发挥对韩合作综合优势，复制和运用自贸试验区改革试点经验，落实中韩自贸协定有关规定，探索对外经济合作新模式、新路径，把中韩（惠州）产业园打造成对外开放的新高地。

　　着力推进贸易强市建设。优化加工贸易、发展一般贸易、提升服务贸易，促进贸易提质发展。落实《发展壮大一般贸易五年行动计划》，支持特色产品创建自主品牌，搭建品牌推广服务平台。大力发展外贸新业态新模式，引进培育外贸综合服务企业，积极申报建设惠州综合保税物流园区，支持企业建设跨境电商海外仓和商品展销中心。加强外贸品牌及质量建设，争取培育认定5家出口品牌企业。实行风险监控和分级分类检验检疫监管模式，对符合规定的企业纳入出口退（免）税一、二类管理。

　　拓展对外交流合作领域。主动对接“一带一路”和广东自贸区建设，积极推动广东铁路国际物流基地建设。加大与周边港口合作力度，努力拓展国际海运航线。推动惠州机场开通国际航线。加快建设海峡两岸青年创业基地、广东惠州留学人员创业园。发挥海外经贸文化联络处、经贸代表处等驻外机构作用，继续推进科技、教育、文化、卫生、旅游等方面的国际交流与合作。推动与英国伍斯特郡等地结为国际友好城市。办好“惠货全球行”印尼雅加达展览会等经贸交流活动。

　　（四）加大改革攻坚力度，推动体制机制焕发新活力

　　深入推进“放管服”改革。加快政府职能转变，承接好省下放的用地审批、经营许可等领域审批权限，更好向市场和基层放权。继续清理政府部门权责清单，推进行政审批标准化规范化建设，进一步“简政放权”“减证便民”。强化市场监管体系建设，全力推行“双随机一公开”“互联网+监管”，推进协同监管、信用监管、智能监管。全面推进“集成办理、一次搞定”政务服务改革，大力推进数字政府建设，完善政务服务大数据库，打通“信息孤岛”，搭建全覆盖、高效能的电子政务体系，让信息多跑路、群众少跑腿。

　　深化行政管理体制改革。支持配合市县监察体制改革。深入推进经济发达镇行政管理体制改革，进一步激发内生动力。深化事业单位分类改革，强化公益属性，推进政事分开、事企分开、管办分离。健全有利于依法独立行使审计监督权的审计管理体制，继续开展城市管理和综合执法体制改革。做好环境监测机构、文化卫生计生综合监督行政执法等专项改革。

　　增创营商环境新优势。全面对标国际一流标准，推进营商环境综合改革，抓好法治化国际化营商环境省级试点。出台促进外资发展政策，进一步扩大外资市场准入，建立健全政策措施、服务支撑和法治保障体系，提升利用外资质量与水平。抓好投资项目审批制度改革，深化公共资源交易体制改革，进一步规范“中介超市”建设。全面推进“证照分离”，完善“多证合一”改革，优化开办企业综合服务平台功能，提升开办企业便利度。加强社会信用体系建设，营造崇商文化氛围，构建“亲”“清”新型政商关系。

　　（五）加快城市更新改造，推动城市发展水平迈上新台阶

　　推进城市精细化管理。完成城市空间发展战略规划和新一轮城市总体规划修编，抓好新城、新区、产业平台和城市更新提质规划研究。开展历史城区保护详细规划，完善历史文化名城保护规划体系。推进“多规合一”，提升规划信息化水平。拓展“和美网格”应用，推进数字城管向智慧城管升级，引领智慧城市建设。健全查处机制，铁腕推进“两违”专项整治。完成第二次全国地名普查、第一次全国地理国情普查工作，全面开展智慧惠州时空大数据与云平台建设试点工作。发展智慧交通，建设TOCC，提高交通协调应急指挥能力。

　　加快“两港三网”建设。建成武深高速、从莞高速惠州段，加快推进河惠莞高速、汕湛高速惠清段、惠龙高速等建设。抓好赣深高铁、广汕高铁惠州段建设，加快惠州北枢纽站建设，积极推进深惠城轨、惠州轨道交通1号线、深圳地铁14、16号线惠州延长线等项目前期工作。加快惠州客运中心站建设，确保惠州汽车客运南站建成投运。完善港口配套设施，建成荃湾煤码头进港铁路等项目。加快惠州机场扩容扩建工程，推进“千万级新干线机场”建设步伐。推动千兆光纤进小区、百兆进家庭，加快5G信息基础设施建设及IPV6推广应用。

　　强化市政设施建设。逐步完善城市公共基本配套设施，加快推进仲恺大道、四环路南段、惠新大道、梅湖大道等项目建设，建成金恺大道、隆生大桥，启动北环大道、金石三路等项目建设。推进城市深地资源和地下空间开发利用试点工作。加快公交专用道建设，建成省级公交示范城市。推动金山湖公园三期建设，建成激流坑等一批公园。加快500千伏演达输变电工程、220千伏维布输变电工程等重点电网项目建设。推进一批生活垃圾、建筑垃圾、餐橱垃圾处理等环卫设施建设。

　　抓好国家“城市双修”试点。编制“生态修复城市修补”专项规划，推动西湖周边老旧小区雨污分流改造、祝屋巷文创街区建设、潼湖湿地公园等8个“城市双修”示范项目。实施望江片及龙丰小新村棚户区改造工程，打造棚改示范性项目。推动金山湖片区、惠博路沿线片区等“三旧”改造项目。开展城市排涝改造综合整治，完成市中心区10处易积水点改造。推进金龙大道地下综合管廊建设，加快金山湖片区“海绵城市”示范区建设。

　　（六）大力实施乡村振兴战略，推动城乡融合发展呈现新面貌

　　扎实推进农业供给侧结构性改革。坚持质量兴农、绿色兴农、效益优先，加快推进农业由增产导向转向提质导向。落实农村土地承包“三权分置”办法，推进农村集体产权制度改革试点，推动农业政策性保险扩面提标。开展优势特色农业提质增效行动，培育知名农产品品牌。创新现代农业经营体制，支持农业龙头企业、专业合作社和家庭农场发展。推进农产品精深加工，培育壮大农产品电商、休闲农业，促进农村一二三产业融合发展。建设省农科院惠州现代农业促进中心，完成市农业综合检测中心主体工程。坚守耕地和基本农田红线，完成7.48万亩高标准基本农田、5000亩垦造水田建设任务。强化粮食保供稳价和储备粮管理，提高粮食安全保障水平。

　　全面推进美丽乡村建设。按照“产业兴旺、生态宜居、乡风文明、治理有效、生活富裕”的总要求，注重“抓两头、带中间”“先达标、再提升”，以“3+3+X”模式统筹新农村建设，加快推动150个省市新农村示范村创建工作。开展“三清理、三拆除、三整治”，确保完成20户以上自然村环境综合整治任务。培育“旅游+农业+景区”新模式，开展农业公园创建工作，打造一批农业观光旅游示范片。鼓励创办乡村旅游合作社，规范民宿发展。大力推动“厕所革命”，补齐影响群众生活品质的短板。建设“四好农村路”，完成2552公里农村公路安全生命防护工程。加强农村公共文化建设，培育文明乡风、良好家风、淳朴民风。

　　加快发展特色小镇。因地制宜、突出特色，高起点编制特色小镇建设实施方案。鼓励重点镇、中心镇、专业镇充分发挥资源禀赋及产业传统优势，做大做强主导产业，优化提升特色产业。坚持“示范一批、创建一批、培育一批”，着力打造休闲旅游、民俗文化、科技教育、健康疗养等30个特色小镇。优化空间布局，强化特色小镇对周边乡村的辐射与带动作用，形成点、线、面联动发展格局。

　　巩固脱贫攻坚成果。深入实施脱贫攻坚八项工程，完善贫困地区交通、电力、通讯、农田水利等基础设施。注重扶贫和扶志、扶智相结合，突出产业扶贫，激活“造血功能”，巩固提升“村村有物业、户户有就业、人人有收入”成果。健全38772预脱贫人口稳定增收长效机制，完善贫困人口动态管理。传承红色基因，加快高潭、安墩等革命老区发展。落实教育、医疗、住房等保障措施，促进低保政策与扶贫政策有效衔接，确保贫困户稳定实现“两不愁、三保障”，确保脱真贫、真脱贫。

　　（七）牢固树立绿色发展理念，推动生态文明建设增添新优势

　　持续推进生态建设。优化生态安全屏障体系，深入开展绿色城乡“六大行动”，巩固提升国家生态文明建设示范市创建成果。扎实开展国家生态园林城市创建，完成国家节水型城市创建省级申报。落实珠三角国家森林城市群建设任务，创建5个省级森林小镇。启动市植物园二期建设。加强生态公益林划定管理。全面完成国有林场改革任务。实施海洋生态文明建设行动计划，推进海岸带整治修复，规范海域海岛使用管理，完成考洲洋美丽海湾、东山海大型人工鱼礁示范区省级试点建设。

　　坚决打好大气、水、土壤污染防治三大战役。深入实施三个“十条”，切实加强污染防治。全面落实“1+2+N”大气污染防治措施，继续强化扬尘及臭氧污染防控，环境空气质量优良率95%以上，坚决守住“惠州蓝”。进一步完善河长制，建立健全湖长制，继续推进“两河”流域和沙河、公庄河等重点流域综合整治，全面完成城市建成区黑臭水体和镇级河涌整治。建成金山污水处理厂二期、马安污水处理厂，完成所有重点流域区域污水处理厂提标升级，完成东江、西枝江沿岸151个自然村污水处理设施建设任务，新建截污管网500公里以上。健全土壤污染防治体系，完成农用地土壤状况详查工作，强化土壤环境分类管控，逐步推进污染地块修复。建成市生态环境园一期垃圾焚烧发电厂。做好第二次全国污染源普查、第一轮省级环保督察迎检工作。

　　推动绿色低碳发展。坚决推行能源和水资源消耗、建设用地等总量和强度“双控”，全面推进各领域减排。抓好企业能源管理中心建设，提高企业用能精细化管理水平。支持企业（园区）积极申报国家绿色制造试点示范。大力推广应用节能低碳环保新技术、新产品，完成绿色建筑、装配式建筑专项规划编制，新建建筑中绿色建筑占比50%以上。推进绿色交通，大力推广新能源汽车。深入创建节约型公共机构示范单位。推进垃圾分类处置利用，促进垃圾处理减量化、资源化、无害化。

　　严格落实环境保护机制。扎实推行自然资源资产清单管理、GDP与GEP“双核算”制度，开展自然资源资产绩效评价，确保生态环境保护责任落实到位。实行生态空间用途管制，严格执行“三个一律不批”和“三个限批”，完善环境监测网络和预警机制，强化污染源头控制。全面实施海洋生态红线和重点海域陆源污染物总量控制制度。全面开展排污企业环保信用评价。健全环保执法机制，严惩环境违法行为。完善生态补偿制度，实现“谁保护、谁受偿”“谁改善、谁得益”。

　　（八）加强风险隐患排查，推动防范化解风险取得新成效

　　积极防范化解金融风险。完善地方金融风险监测防控机制，加强金融监管协调和数据共享，强化对金融风险的监测预警和应急处置。严格规范实体经济企业从事金融业务，大力遏制资金“脱实向虚”。大力打击非法集资，重点规范互联网金融，加强对涉众金融、国企债务的预估研判，做好12.20事件等问题的处置工作，坚决守住不发生区域性金融风险的底线。

　　严控政府债务风险。坚决落实地方政府债务限额管理要求，确保不突破债务限额。全力遏制隐性债务增量，摸清地方政府债务情况，特别是核实以PPP、明股实债、违规担保等方式变相举债情况，稳妥处置具有较高风险的隐性债务。

　　促进房地产市场健康发展。牢牢把握“房子是用来住的、不是用来炒的”定位，加快建立多主体供给、多渠道保障、租购并举的住房制度。加快培育和发展住房租赁市场，支持专业化、机构化住房租赁企业发展。严格落实房地产市场调控政策，严格限制信贷资金用于投资投机性购房，坚决防范房地产市场引发金融风险。

　　切实维护公共安全。完善公共安全风险分类分级管控制度，建设市应急指挥平台。严格落实安全生产责任制，加强安全生产监管执法，铁腕整治事故隐患，有效防范和坚决遏制较大以上生产安全事故。积极创建省级食品安全城市，重点加强学校、农村、网络订餐食品安全和食品加工小作坊专项整治，确保群众“舌尖上的安全”。提升“三防”实时监测、应急指挥能力，做好气象预警预报工作，建设“平安海洋”。

　　强化平安惠州建设。推动社会治理重心下移，加强城乡社区精细化治理，深化网格化管理。加快推进 “雪亮工程”，打造社会治安“智慧防控圈”。严密防范和坚决打击各种暴力活动，持续开展打击黄赌毒黑拐骗等违法犯罪专项行动，深入推进全民禁毒工程。强化信息网络安全防控，着力解决电信诈骗、倒卖个人信息等问题。健全公共法律服务体系，推进“三社”联动。加强综治信访维稳工作，建立健全社会矛盾纠纷滚动排查化解机制。

　　（九）坚持以人民为中心的发展思想，推动社会民生事业再上新水平

　　全力办好民生实事。坚持把增进民生福祉作为政府一切工作的出发点和落脚点，始终把百姓冷暖、群众利益记在心上。投入19亿元，集中力量办好十件17项民生实事（具体项目见附件），不折不扣落实好省民生实事涉及我市的相关项目，确保全面完成任务。

　　优先发展教育事业。围绕建设广东省推进教育现代化标兵市，落实发展学前教育第三期行动计划实施方案，鼓励社会力量兴办普惠性幼儿园，力争新增学前教育学位2万个。加大基础教育学位供给，力争新增义务教育学位2.4万个。加快“智慧教育”建设，着力解决中小学生课外负担重等突出问题。深入实施高中阶段教育普及攻坚计划，确保惠州中学如期开学。推进义务教育现代化学校建设，抓好10个省教育强镇复评。完善扶持激励机制，支持规范民办教育发展。深化产教融合、校企合作，推动职业教育做优做强，建成惠州工程职业学院三期。支持惠州学院建设理工科特色鲜明的高水平应用型大学。落实科交会成果，推动国内外高校来惠建设研究院和新校区。

　　加快健康惠州建设。深化医药卫生领域综合改革，加快卫生与健康“十大工程”建设。完善“三医”融合、基层“两生整合”，全面推进医联体建设。构建分级诊疗新格局，大力推广家庭医生签约服务。加快杨村、多祝中心卫生院建设，推进7家镇级卫生院升级改造，完成175间省级村卫生站标准化建设。落实全科医生倍增计划，抓好基层卫生人才培养。加快实施名科、名馆、名医、名产品、名基地等“五名”工程，积极引进具有省级以上领先水平的医疗学科团队。深入实施“互联网+健康医疗”工程，推进医疗健康大数据广泛应用。探索构建全民健康服务体系，免费对全市常住人口实施体检，免费为本市户籍60周岁及以上老年人接种流感和肺炎疫苗。大力传承发展中医药事业，积极申报全国中医药综合改革试验区。实施妇幼健康工程，提高基本公共卫生计生服务均等化水平。广泛开展爱国卫生运动，推动省健康城市试点工作，做好国家卫生城市迎复审。

　　促进文化体育事业繁荣发展。深入推进文化强市建设，抓好市美术馆、规划馆等公共文化场馆建设。新建262个综合文化服务中心，实现村（社区）全覆盖。持续实施文化惠民工程，推出一批具有惠州特色、体现时代精神、传递正能量的好作品。强化历史建筑、特色街区保护修缮和科学利用，建立历史文化名城信息管理系统。扎实抓好国家文化消费试点工作，争创全国文化消费示范市。推进“文化+”战略，加快惠阳吉他小镇、罗浮山影视文化产业基地、赛格国际动漫产业基地、奥飞南昆山星湖小镇等文化产业项目建设。持续推进“版权兴业”工程，巩固机关企业软件正版化工作成果。基本实现农村广播电视无线数字信号全覆盖。承办好第四届粤港澳台微影视作品文化交流周。开展公民文明素质和社会文明程度提升行动，积极培育和践行社会主义核心价值观，深化“好人之城”“志愿之城”建设，巩固提升文明创建成果。举办500场以上全民健身活动，承办好中华龙舟大赛、全国青年花样游泳锦标赛，做好省第六届少数民族运动会备战和十五届省运会参赛工作。

　　完善多层次社会保障体系。坚持兜底线、织密网、建机制，保障和改善民生。实施更加积极的就业政策，力争新增城镇就业6.5万人，创业带动就业1.7万人，转移农村富余劳动力8000人。落实企业职工养老保险省级统筹，积极推进社会基本医疗保险城乡一体化，加强基层社保经办服务能力建设。进一步改革医保支付方式，完善多种付费方式并存的复合支付方式。继续完善工伤保险政策制度，试点推广工伤医疗待遇实时结算。提高低保、孤儿、特困人员生活保障标准。推进“一站三中心”建设，完善社区和居家养老体系，发展社会福利和慈善事业。做好双拥优抚安置保障工作，完善复退军人服务体系。推动老旧小区改造，鼓励老旧住宅楼加建电梯。加强农村住房建设技术指导，完成农村危房改造任务。

　　继续加强国防动员和后备力量建设，扎实做好援藏援疆援川和对口城市合作等工作，积极发展妇女儿童、青少年、老年人、残疾人、红十字等事业，着力抓好民族宗教、防震减灾、消防、应急、移民、统计、科普、方志、档案、侨务等工作，推动社会事业全面进步。

　　（十）始终以人民满意为目标，推动政府自身建设实现新提升

　　旗帜鲜明讲政治。坚定不移落实“党领导一切”的根本原则，牢固树立“四个意识”，坚决维护以习近平同志为核心的党中央权威和集中统一领导，始终在思想上政治上行动上同以习近平同志为核心的党中央保持高度一致。认真贯彻落实省委、省政府的决策部署，坚决维护市委总揽全局、协调各方的领导作用，确保政令畅通、令行禁止。严格遵守党章和党内政治生活准则，坚决落实民主集中制，自觉做到守纪律、讲规矩。

　　加快建设法治政府。深入贯彻落实新时代全面依法治国基本方略和法治政府建设实施纲要，积极参与珠三角法治政府示范区创建工作。落实集体学法、法律顾问、决策咨询等制度，做好政府立法工作。健全普法宣传教育机制，落实“七五”普法规划和“谁执法谁普法”普法责任制。规范公正文明执法，严格落实行政执法责任制。自觉接受人大依法监督、政协民主监督和监察监督、司法监督，切实加强审计监督，主动接受社会监督、舆论监督，确保行政权力在阳光下运行。

　　打造廉洁高效政府。扎实开展“不忘初心，牢记使命”主题教育，全面贯彻中央八项规定精神及实施细则，驰而不息纠正“四风”。坚持政府过“紧日子”，让企业和群众过“好日子”，严格预算管理，优化支出结构，提高财政资金使用绩效。坚定推进全面从严治党，全力支持纪检监察机关监督执纪问责。坚持把狠抓落实、有效执行作为政府工作的生命线，深入开展“大学习、深调研、真落实”活动，切实增强群众观念、群众感情，强化服务意识、效率意识，“马上就办、真抓实干”，确保事事有落实、件件有回音。

　　各位代表！实干创未来，筑梦谱新篇。让我们高举习近平新时代中国特色社会主义思想伟大旗帜，更加紧密团结在以习近平同志为核心的党中央周围，按照习近平总书记对广东工作的重要指示批示精神，在省委、省政府和市委的坚强领导下，不忘初心、牢记使命，以永不懈怠的精神状态和一往无前的奋斗姿态，始终同全市人民想在一起、干在一起，不断谱写建设绿色化现代山水城市新篇章，为实现人民群众对美好生活的向往而努力奋斗！

　　附件：

　　惠州市2018年十件民生实事

　　（一）实施健康惠民项目。开展全民预防保健工作，对全市常住人口实施免费体检（其中0-6周岁及65周岁以上人群每年享受一次免费体检，其他年龄段人群每三年享受一次免费体检）和健康管理服务，进一步增强全民预防保健意识，提升我市居民健康水平，推进健康惠州建设；通过基本医疗保险保障、大病保险二次补偿、城乡医疗救助托底、慈善医疗救助补充相结合的方式，实现全市户籍贫困人口（含低保、五保、城镇三无、孤儿、建档立卡扶贫等对象共约8万人）县域内住院政策内基本医疗免费，进一步解决因病致贫、因病返贫问题。免费为本市户籍60周岁及以上老年人接种流感和肺炎疫苗（流感疫苗82元/剂，一年一次；肺炎疫苗217元/剂，五年一次），提高老年人健康水平。

　　（二）加大对严重精神障碍患者保障力度。对高风险严重精神障碍患者的监护人和协助监护人分别按每人每月450元和100元标准给予补助，一般性精神障碍患者的监护人按每人每月200元标准补助，加强严重精神障碍患者管理及救助救治工作，将贫困重性精神疾病患者住院治疗标准由1600元/月提高到1750元/月，免费发药标准由50元/月提高到70元/月。

　　（三）提高居民抵御风险能力。惠州市自然灾害和公共突发事件公众责任保险保障范围扩大至保险责任事故发生时处于惠州市行政区域内的所有自然人，增强居民应对自然灾害和公共突发事件能力。老年人意外伤害综合保险的受益人群从本市户籍70周岁以上老年人扩大到本市户籍60周岁以上的老年人，提升居民抵御意外伤害风险能力。

　　（四）推进乡村振兴战略。按照“产业兴旺、生态宜居、乡村文明、治理有效、生活宽裕”总要求，全力实施150个省市贫困村创建社会主义新农村示范村，深化“3+3+X”的建设模式，实现创建示范村达到整治标准，切实改善农村的生产生活生态条件，巩固扶贫攻坚的成果，基本建成4个省级新农村示范片，全面启动社会主义新农村建设，完善整治规划，完成“三清三拆三整治”。市对县（区）农村保洁员补助提高10%，即市对县农村保洁员每人每月补助达到660元，市对区农村保洁员每人每月补助达到550元，调动保洁员服务农村积极性。

　　（五）健全农产品质量安全监管体系。按照省级农检机构的标准建设市农业综合检测中心，实现对农业产出品、投入品和产地环境检测全覆盖，加强农产品质量监测和监督抽查，全面排查农产品安全隐患，对重点环节进行专项检查，切实保障“从农田到餐桌上”的农产品质量安全，为强化农产品质量安全监管体系，完善农业服务体系等工作提供有力的技术支撑。

　　（六）优化教育教学条件。对全市范围内在符合普惠性收费标准的民办幼儿园就读的儿童按每生每年400元标准给予补助，继续提升办园水平和保教质量。推进惠州中学工程项目建设，缓解学位紧缺、班额大问题。

　　（七）提高文化惠民水平。利用“文惠网”向市民发放文化消费补贴电子消费券，打造系列文化消费季活动，带动市民参与文化消费。实施慈云图书馆100万册纸质图书增量计划，为市民提供更多阅读和学习的选择。

　　（八）实施东江、西枝江生态保护工程。实施东江、西枝江干流沿岸自然村生活污水处理设施全覆盖，151个自然村建设生活污水设施，在现有奖补政策基础上，对2018年建成的污水处理设施给予每座10万元的奖励，加快提升污水处理设施治污减排效益，确保东江饮用水源安全。加强西枝江流域生态保护，对西枝江流域生态保护进行专项资金补偿，改善西枝江流域生态环境质量。

　　（九）提升公交服务水平。加快公交体制机制改革，惠城区公交行业统一实行TC模式运营和管理，对惠州机场客运专线实行公交化改造，新增及优化调整公交线路20条，新增及更新纯电动新能源车258辆，升级改造惠城区公交简易站牌500个，推出乘坐公交电子移动支付便民服务，提升公交出行便利性、环保性和安全性。

　　（十）缓解交通压力提高行人交通安全。推进隆生大桥桥梁、道路及排水管道等工程项目施工，完成隆生大桥及其连接道路工程建设，缓解市中心交通拥堵压力。实施行人过街一体式信号灯交通设施安装项目，在市区主干道人行横道线处设置行人过街一体式信号灯，方便市民横过马路，提高安全性。
